# Comparative Signature-Tagged Mutagenesis Identifies *Pseudomonas* Factors Conferring Resistance to the Pulmonary Collectin SP-A

**DOI:** 10.1371/journal.ppat.0010031

**Published:** 2005-11-25

**Authors:** Shiping Zhang, Yi Chen, Eric Potvin, Francois Sanschagrin, Roger C Levesque, Francis X McCormack, Gee W Lau

**Affiliations:** 1 Division of Pulmonary and Critical Care Medicine, University of Cincinnati College of Medicine, Cincinnati, Ohio, United States of America; 2 Centre de Recherche sur la Fonction Structure et Ingenierie des Proteines, Universite Laval, Ste-Foy, Quebec, Canada; Harvard Medical School, United States of America

## Abstract

The pulmonary collectin, surfactant protein A (SP-A), is a broad spectrum opsonin with microbicidal membrane permeabilization properties that plays a role in the innate immune response of the lung. However, the factors that govern SP-A's microbial specificity and the mechanisms by which it mediates membrane permeabilization and opsonization are not fully understood. In an effort to identify bacterial factors that confer susceptibility or resistance to SP-A, we used comparative signature-tagged mutagenesis to screen a library of 1,680 *Pseudomonas aeruginosa* mutants for evidence of differential pulmonary clearance in SP-A-sufficient (SP-A^+/+^) and SP-A-deficient (SP-A^−/−^) mice. Two SP-A-sensitive *P. aeruginosa* mutants harboring transposon insertions in genes required for salicylate biosynthesis *(pch)* and phosphoenolpyruvate-protein-phosphotransferase *(ptsP)* were recovered. The mutants were indistinguishable from the parental wild-type PA01 with regard to opsonization by SP-A, but they exhibited increased susceptibility to SP-A-mediated membrane permeabilization. These results suggest that bacterial gene functions that are required to maintain membrane integrity play crucial roles in resistance of *P. aeruginosa* to the permeabilizing effects of SP-A.

## Synopsis

Everyday, normal breathing deposits numerous microorganisms on the surfactant membrane that lines the air-exchanging surfaces of the lung. Surfactant protein SP-A, a component of the surfactant membrane, helps to maintain the lung in a germ-free state by aggregating inhaled microorganisms and facilitating their ingestion by immune cells, and by increasing the permeability of their cell membranes. However, the bacterial pathogen *Pseudomonas aeruginosa* is resistant to SP-A-mediated membrane disruption.

Using a genetic tool called comparative signature-tagged mutagenesis, the authors have identified two *P. aeruginosa* genes, *pch* and *ptsP,* that are required to resist SP-A-mediated membrane permeabilization. Molecular analyses indicate that the *pch* gene encodes an enzyme that synthesizes salicylate, a compound utilized by bacteria to acquire essential metal ions. The *ptsP* gene encodes an enzyme called phosphoenolpyruvate-protein-phosphotransferase. The loss of salicylate and phosphoenolpyruvate-protein-phosphotransferase weakens the *P. aeruginosa* cell membrane, which allows SP-A to poke holes on the membrane and kill the bacteria.

This is the first known report of the roles played by salicylate and phosphoenolpyruvate-protein-phosphotransferase in maintenance of bacterial membrane, and consequently, protecting bacteria from killing by SP-A, through disruption of membrane integrity.

## Introduction

Pulmonary surfactant is a protein-phospholipid complex that lines the air-liquid interface of alveoli, forming the first point of contact with inhaled microbial pathogens [1,2]. A multitude of in vitro studies have demonstrated that the pulmonary collectins, surfactant protein A (SP-A) and surfactant protein D (SP-D), bind and aggregate bacterial, fungal, viral, and mycobacterial organisms, directly activate macrophages, and enhance the in vitro phagocytosis and intracellular killing of a variety of pulmonary pathogens [3,4]. Recognition of diverse microbial species by the pulmonary collectins is mediated by the C-type lectin domain, which selectively binds to carbohydrates that are prevalent on the surface of microbes, but not to the predominant terminal sugars on surface molecules of mammalian cells [5]. More recently, SP-A and SP-D have been reported to possess potent antimicrobial properties [6–8]. They directly inhibit the proliferation of bacteria and fungi in a macrophage- and aggregation-independent manner, by increasing the permeability of the microbial cell membrane. In particular, rough strains of Gram-negative bacteria, containing truncated lipopolysaccharide (LPS) domains are uniquely vulnerable to permeabilization by the collectins [6], as has been well known for other antimicrobial peptides and proteins.

Although the collectins inhibit microbial growth, the factors that govern the specificity and the mechanisms of membrane disruption by the collectins are not understood. In this study, we employed comparative signature-tagged mutagenesis (STM) as a tool to identify bacterial factors required to resist SP-A-mediated clearance, in vivo. STM relies on the ability of the pathogen in question to replicate in vivo as a mixed population and allows for the identification of the mutants whose phenotypes cannot be trans-complemented by other virulent strains present in the same inoculum [9]. Two *Pseudomonas* factors that are specifically required to protect *P. aeruginosa* from killing by SP-A were identified and characterized.

## Results

### SP-A^−/−^ Mice Are Susceptible to Infection by *P. aeruginosa*

**Figure 1:**
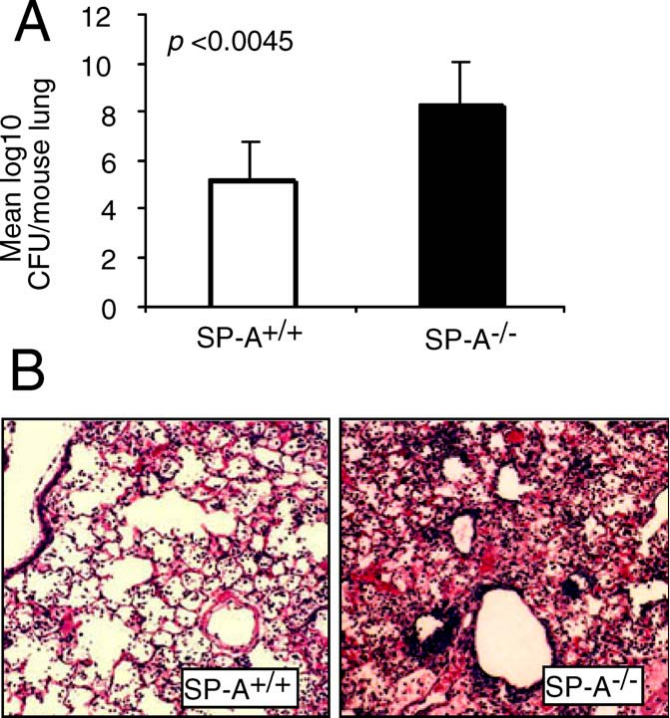
SP-A Deficient Mice Are More Susceptible to Infection by *P. aeruginosa* (A) SP-A^+/+^ and SP-A^−/−^ mice (group of six) were infected intranasally with 1 × 10^7^ *P. aeruginosa* PA01. Bacteria were recovered from homogenized lung tissue 16 h after inoculation. *p* < 0.01. (B) Representative lung histology sections (stained with hematoxylin and eosin) from SP-A^+/+^ and SP-A^−/−^ mice 16 h post intranasal instillation of PA01. Original magnification: 10 ×.

Levine et al [10] have reported that outbred SP-A^−/−^ mice exhibit delayed clearance of a clinical *P. aeruginosa* strain from the lung, suggesting that SP-A plays a role in pulmonary innate immunity against this organism. We also found that the inbred C3H/HeN SP-A^−/−^ mice were more susceptible to *P. aeruginosa* infection, in this case by strain PA01. SP-A^+/+^ mice cleared 63-fold (1.8 log) more of intranasally inoculated PA01 by 16 h (Figure 1A). In contrast, the bacterial load increased by 22 fold (1.34 log) in SP-A^−/−^ mice (Figure 1A). In the absence of infection, SP-A^−/−^ mice are healthy, and the histopathological analyses of their non-infected lungs revealed no significant differences when compare to the lungs of SP-A^+/+^ mice (unpublished data). When infected with PA01 however, SP-A^+/+^ mice developed broncho-pneumonia with mild pulmonary infiltrates (Figure 1B). In contrast, SP-A^−/−^ mice developed more extensive consolidation with areas of lobar pneumonia.

### Identification of *P. aeruginosa* Genes Conferring Resistance to SP-A by Comparative STM Screening

**Figure 2:**
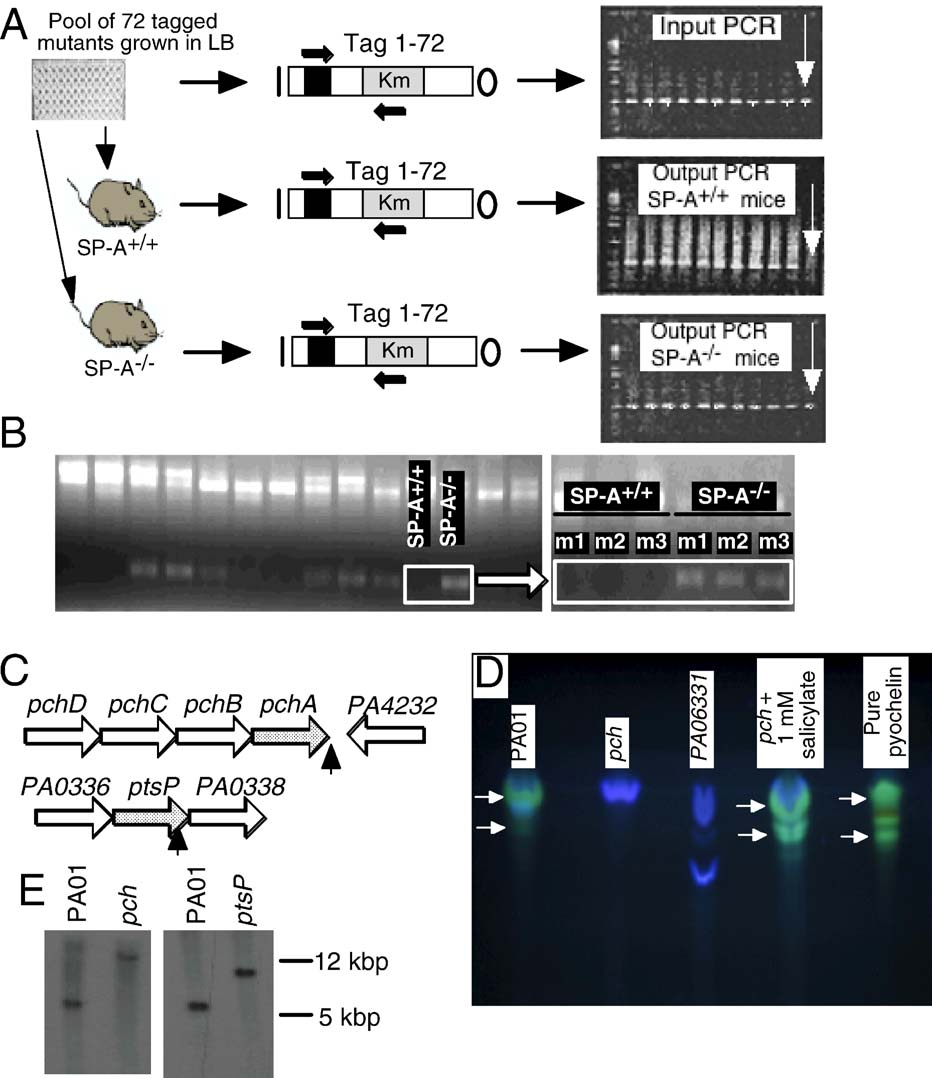
PCR-Based Signature-Tagged Mutagenesis (A) Schematic drawing of PCR-based STM. Pools of 72 uniquely tagged mutants were intranasally inoculated into SP-A^+/+^ and SP-A^−/−^ mice; 16 h later, lungs were harvested, homogenized, and plated. Approximately 10,000 colonies were harvested from the plates for genomic DNA extraction. PCR-amplification of tags was performed on the genomic DNA to screen for the presence or absence of DNA tags of each of the 72 mutants. Mutants whose DNA tags were present in the input pool and in the SP-A^−/−^ pool, but absent in the SP-A^+/+^ pool (see white arrows) were further screened for susceptibility to SP-A. (B) Agarose gel of PCR-based STM, identifying the *pch* mutant (left panel). Attenuation in the first SP-A^+/+^ mouse (right panel, m1) was confirmed in two additional SP-A^+/+^ mice (right panel, m2 and m3). (C) Genetic loci of *P. aeruginosa,* when mutated, conferred increased sensitivity to in vivo killing by SP-A. DNA regions flanking pUTmini-Tn*5* transposon insertions were cloned and sequenced. Similarity BLAST searches were performed against *P. aeruginosa* PA01 genomic sequence on NCBI and on http://www.pseudomonas.com. Black arrows indicate the approximate insertion site within the mutated ORFS. (D) TLC analyses indicate that the *pch* STM mutant is unable to synthesize pyochelin (see arrows). Wild-type PA01 grown in LB and the *pch* mutant grown in LB supplemented with 1 mM salicylate produced green color pyochelin (see arrows). In contrast, no observable pyochelin was produced by the *pch* and *PA06331* strains. Pure pyochelin was used as control. (E) Restriction fragment length polymorphism analysis between parental wild-type PA01 and STM mutants confirmed that mutations in *pch* and *ptsP* were caused by a single insertion.

We exploited the PCR-based STM technique and the *P. aeruginosa* PA01 STM mutant library [11] to identify putative microbial targets for SP-A. SP-A^+/+^ and SP-A^−/−^ mice, in groups of three, were intranasally challenged with pools of 72 oligo-tagged STM mutants. Lungs were harvested at 16 h, homogenized, and plated. At least 10,000 bacterial colonies grown on Luria broth (LB) agar plates were harvested from each lung for bacterial genomic DNA extractions and oligo-tag amplification. We screened for differential clearance of individual STM mutants using PCR. If the PCR product of a mutated gene was absent on the output gel in SP-A^+/+^ mice, but present in SP-A^−/−^ mice (Figure 2A, see arrows), we postulated that the bacterial protein encoded by the mutated gene was required to overcome the host defense activities of SP-A.

We comparatively screened 1,680 STM mutant strains in SP-A^+/+^ and SP-A^−/−^ mice. Two mutants with increased sensitivity to SP-A (i.e., failed to survive in SP-A^+/+^ mice) were recovered, for a 0.12% recovery rate. We have cloned and sequenced both STM mutants by plasmid rescue [11]. DNA sequences were analyzed by comparison with online databases (http://www.pseudomonas.com). The output from Pool 4 (mutant *pch*, see below) is shown in Figure 2B (left panel). In the presence of SP-A, the *pch* mutant, which is disrupted in a gene required for salicylate biosynthesis, was unable to survive in the SP-A^+/+^ lung (missing band in the box, Figure 2B). In absence of SP-A, the *pch* mutant was able to grow in the lung, as indicated by the presence of an oligo-tag amplified by PCR (Figure 2B, left panel, SP-A^−/−^ lane; right panel, lane m1). The results were confirmed by PCR analysis of the output pool of two additional mice (Figure 2B, right panel, lanes m2 and m3).

The *pch* STM mutant carries a transposon pUTminiTn*5*Km2 insertion within an intergenic region in between the 10^th^ and 11^th^ nucleotide after the stop codon of the *pchA (PA4231)* gene (Figure 2C). The *pchA* gene encodes an isochorismate synthase that participates in the biosynthesis of the siderophores, salicylate, and pyochelin [12–14]. Because transposon inserted within the intergenic region immediately downstream of the *pchA* gene, it might have affected the mRNA stability of the *pchDCBA* transcript. Thus, we named the STM mutant as *pch*. We examined whether the *pch* mutant had lost the ability to synthesize pyochelin by using thin layer chromatography (TLC) assays. TLC analysis of pyochelin extracted from bacteria cultured in M63 minimal medium confirmed that the *pch* mutant is unable to synthesize the green-colored pyochelin (Figure 2D, *pch* lane). In contrast, the parental wild-type PA01 produced pyochelin (Figure 2D, PA01 lane). When the *pch* STM mutant was grown in the presence of 1 mM salicylate, however, the ability to synthesize pyochelin was restored (Figure 2D, *pch* + 1mM salicylate lane). The expression of the genes within the downstream operon *(PA4232* and *PA4233)*, which is transcribed in opposite orientation to the *pchADCBA* operon, was found to be unaffected by transposon insertion as assessed by mRNA expression analyses (unpublished data). Collectively, these data suggest that susceptibility to SP-A-mediated clearance is caused by the loss of *pch* gene function, most probably by affecting the mRNA stability of the *pchDCBA* operon.

The second SP-A-susceptible STM mutant had the transposon pUTminiTn*5*Km2 inserted in between the 2,213^th^ and 2,214^th^ nucleotides of the *ptsP (PA0337)* gene (Figure 2C). The *ptsP* gene encodes a phosphoenolpyruvate-protein-phosphotransferase, a homolog of the *E. coli* Enzyme I^nitrogen^ (EI^Ntr^) [15]. The transposon pUTminiTn*5*Km2 insertion into *ptsP* is predicted to cause polarity and interrupt the transcription of *PA0338*.

Both STM mutants were judged to harbor a single transposon pUTminiTn*5*Km2 insertion after hybridization with the transposon vector (unpublished data). DNA fragments that flanked both ends of the transposon integrations in *pchA* and *ptsP* genes were cloned from the wild-type PA01. They were used to confirm gene interruption by the integrating transposon, by comparing restriction fragment length polymorphisms (RFLP) between wild-type PA01 and the STM mutants. As shown in Figure 2E, transposon-flanking DNA probes hybridized to DNA fragments with higher molecular weights in the chromosomes of both the *pch* and *ptsP* mutants than their parental strain PA01, indicating transposon insertion.

### SP-A-Sensitive STM Mutants Are as Virulent as Parental Wild-Type in SP-A^−/−^ Mice

**Figure 3:**
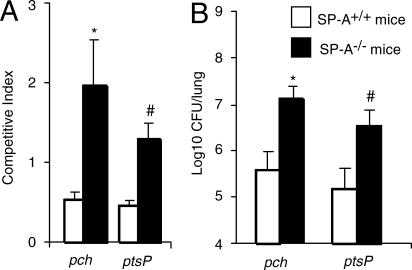
SP-A-Sensitive *P. aeruginosa* STM Mutants Compete Effectively with Parental Wild-Type PA01 in SP-A^−/−^ , but Not in SP-A^+/+^ Mice (A) In vivo competition assays between the wild-type and individual STM mutants after intranasal pulmonary inoculation of 6-wk-old SP-A^+/+^ and SP-A^−/−^ mice. The mean CFU recovered from four mice in each group is shown. The CI is defined as the output ratio of mutant to wild-type bacteria divided by the input ratio of mutant to wild-type bacteria. *p < 0.013, #*p* < 0.011. (B) Single respiratory tract infection of *pch* and *ptsP* mutants in SP-A^+/+^ and SP-A^−/−^ mice was performed as described for Figure 1. Attenuation is defined as the log_10_ difference in CFU between wild-type and mutant bacteria recovered from lung tissue 16 h after inoculation. The mean ± standard error of six mice is shown. *p < 0.019, #*p* < 0.045.

Apart from confronting pulmonary host defenses, our STM screens require each mutant to compete with 71 other STM mutants within individual library pools. Competitive mixed infection assays have been widely used to assess the fitness of individual mutants versus their parental strains during in vivo infection [16,17]. As shown in Figure 3A, when co-administered with the parental wild-type PA01 by intranasal inoculation into mice, both STM mutants were less competitive than PA01 in SP-A^+/+^ mice. Specifically, mutant strains *pch* and *ptsP* were only 53% and 46% as competitive as PA01, respectively. In contrast, both mutants performed equally well as, if not better than, the wild-type strain in SP-A^−/−^ mice (Figure 3A).

We further tested the mutants, *pch* and *ptsP,* in single infection studies. When infected singly into wild-type mice, the viable bacterial counts of *pch* and *ptsP* mutant bacteria were 32-fold (1.5 log) and 25-fold (1.4 log) higher, respectively, in SP-A^−/−^ mice than in SP-A^+/+^ mice (Figure 3B). Our results indicate that SP-A plays an important role in clearance of PA01 mutants with defective *pch* or *ptsP* gene function.

### Preferential Clearance of the STM Mutants from SP-A^+/+^ Lung Is Independent of Macrophages

**Figure 4:**
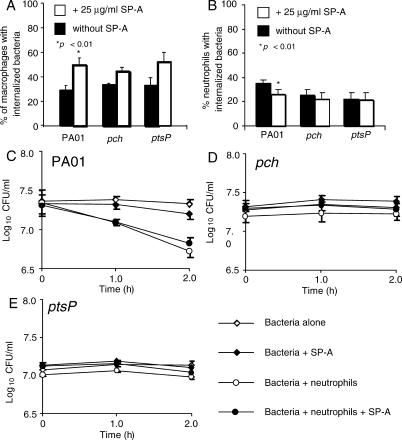
Bacterial Opsonization by SP-A Does Not Result in Differential In Vivo Phagocytosis and Killing of STM Mutants (A) In vitro phagocytosis assays were performed using live, GFP-expressing PA01 and isogenic *pch and ptsP* bacteria, with alveolar macrophages isolated from SP-A^+/+^ mice*.* Internalized bacteria were counted using a phase contrast fluorescence microscope. The means of three experiments are shown. Two hundred macrophages were counted for each mouse. *p* = 0.0058 (PA01 vs PA01 + SP-A), *p* = 0.075 (*pch* vs *pch +* SP-A), and *p* = 0.109 (*ptsP* vs *ptsP +* SP-A). (B) In vitro phagocytosis of PA01, *pch,* and *ptsP* by human neutrophils isolated from healthy volunteers. Phagocytosis experiments were performed as described for (A). *p* = 0.037 (PA01 vs PA01 + SP-A), *p* = 0.577 (*pch* vs *pch +* SP-A), and *p* = 0.919 (*ptsP* vs *ptsP +* SP-A). (C–E) SP-A did not affect uptake or killing of STM mutants. PA01 (C), but not the STM mutants *pch* (D) and *ptsP* (E) were killed in significant amounts by neutrophils, independent of SP-A. The mean of three separate experiments is shown. *p* < 0.001 when comparing PA01 versus PA01 + neutrophils, and when comparing PA01-SP-A versus PA01-SP-A + neutrophils.

Previous studies have suggested that SP-A opsonizes *P. aeruginosa* and increases uptake by macrophages [10,18]. To determine if SP-A-mediated opsonization contributes to the early clearance of STM mutants, we compared the phagocytosis of *pch* and *ptsP* to PA01 in the presence and absence of SP-A. When live, green fluorescence protein (GFP)-expressing bacteria were exposed to mouse alveolar macrophages in the presence of SP-A, the uptake of PA01, its *pch,* or *ptsP* mutant was increased by 1.8, 1.3, and 1.6 fold, respectively, over that which occurred in the absence of SP-A (Figure 4A). However, the SP-A mediated increase in opsonization was only statistically significant in PA01 *(p<0.01).* The magnitude of the increase in the uptake of PA01 in the presence of SP-A was similar to previously published reports [10,18]. Although the addition of SP-A increased the macrophage uptake of STM mutants, enhancement of phagocytosis was slightly lower than that of wild-type PA01. Therefore, it does not appear that SP-A-mediated opsonization is responsible for the preferential clearance of *pch* and *ptsP* mutants from SP-A^+/+^ mice.

### Preferential Clearance of the STM Mutants in SP-A^+/+^ Lung Is Independent of Neutrophils

Neutrophils are known to play a major role in protecting the lung against bacterial infection. We examined whether bacterial phagocytosis and killing by neutrophils might have led to preferential clearance of *pch* and *ptsP*. As shown in Figure 4B, the uptake of PA01 and the *pch* and *ptsP* mutants was not increased in the presence of SP-A. In fact, SP-A-opsonized PA01 bacteria were phagocytosed less efficiently by neutrophils than untreated bacteria. Interestingly, only wild-type PA01 bacteria were susceptible to killing by neutrophils, with approximately 73.7% killed in the absence of SP-A, and 57.2% killed in the presence of SP-A (Figure 4C). In contrast, both *pch* and *ptsP* mutants were not susceptible to neutrophil killing (Figure 4D and 4E). These results indicate that neutrophils kill *P. aeruginosa* by using a mechanism independent of SP-A opsonization, and lend support to the notion that neutrophils play a less critical role in the preferential clearance of *pch* and *ptsP* mutants from the SP-A^+/+^ mice than other anti-pseudomonal mechanisms.

### The Loss of *pch* and *ptsP* Gene Functions Render *P*. *aeruginosa* Susceptible to SP-A-Mediated Membrane Permeabilization

**Figure 5:**
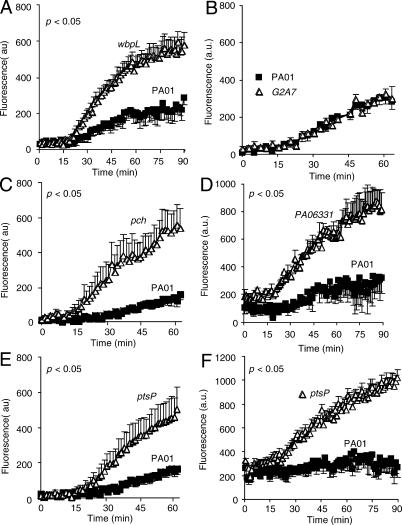
The *pch* and *ptsP* Mutants Are Preferentially Permeabilized and Killed by SP-A (A) Fluorescence of a cleavage-activated phosphatase substrate ELF97 was examined in the presence or absence of 50 μg hSP-A for a period of 60–90 min. Human SP-A (hSP-A) preferentially permeabilized LPS mutant *wbpL,* but not the parental wild-type PA01. The *wbpL* strain was used as positive control for membrane permeability. The difference in membrane permeabilization between *wbpL* mutant and PA01 was significant from the 28^th^ min onward. *p < 0.05. (B) A mutant strain *STMG2A7* that is virulent in both SP-A^+/+^ and SP-A^−/−^ mice was as resistant to SP-A-mediated membrane permeabilization as the parental wild-type PA01. (C–D) Membrane permeability of the STM mutant *pch* and *PA06331*, a *P. aeruginosa* strain which is deleted in all of the structural genes required for pyochelin biosynthesis (Table 1.). The difference in membrane permeabilization between the mutants against wild-type PA01 was significant from the 35^th^ and 18^th^ min onward for mutant strains *pch* and *PA06331*, respectively. *p < 0.05. (E–F) Membrane permeability of the STM mutant *ptsP* and the Δ*ptsP,* a *P. aeruginosa* strain with a nonpolar, inframe deletion of the *ptsP* gene. The difference in membrane permeabilization between the mutants against wild-type PA01 was significant from the 29^th^ and 28^th^ min onward, for mutant strains *ptsP* and Δ*ptsP*, respectively. *p < 0.05.

Recent studies have indicated that SP-A is capable of directly killing bacteria and fungi by membrane permeabilization and inhibition of macro-molecular synthesis, independent of macrophage-mediated phagocytosis [6,7]. We examined whether direct membrane permeabilization contributed to the clearance of the SP-A-sensitive mutants. Due to the previously described importance of LPS in resistance to SP-A-mediated membrane permeabilization [6,19], we used a LPS mutant *wbpL*, which is deficient in the production of “initial” glycosyltransferase essential for the biosynthesis of both the A-band and B-band O-antigen of LPS [20], as positive control for membrane permeabilization assays. Permeabilization of the *wbpL* mutant was approximately 2.7-fold greater than the wild-type strain PA01 at 60 min (Figure 5A). In contrast, *STMG2A7*, an irrelevant mutant that is virulent in both SP-A^+/+^ and SP-A^−/−^ mice, was found to be as resistant as parental wild-type PA01 to SP-A-mediated membrane permeability (Figure 5B).

**Table 1:**
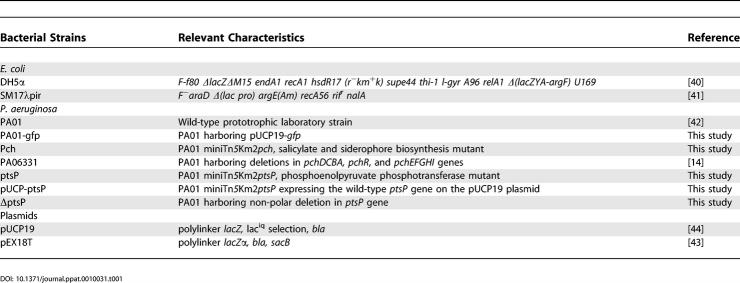
Bacterial Strains and Plasmids Used in this Work

The STM mutant *pch*, and a mutant strain, *PA06331,* in which the pyochelin biosynthetic genes encompassing *pchDCBA*, *pchR*, and *pchEFGHI* operons had been removed [14], were permeabilized by SP-A to an extent that was 3.4- and 2.55-fold greater than the wild-type strain PA01, respectively (Figure 5C and 5D) after 60–90 min exposure to SP-A. Similarly, STM mutant *ptsP*, and an in-frame, nonpolar deletion mutant, Δ*ptsP,* were permeabilized by SP-A to an extent that was 3.0- and 3.8-fold greater than the wild-type strain PA01, respectively (Figure 5E and 5F), at 60–90 min post-exposure to SP-A. These data are comparable to the approximately 3-fold increase in membrane permeability that SP-A exposure induces in *E. coli* K12 [6].

### SP-A-Mediated Membrane Permeabilization Directly Kills the *pch* and *ptsP* Mutants

**Figure 6:**
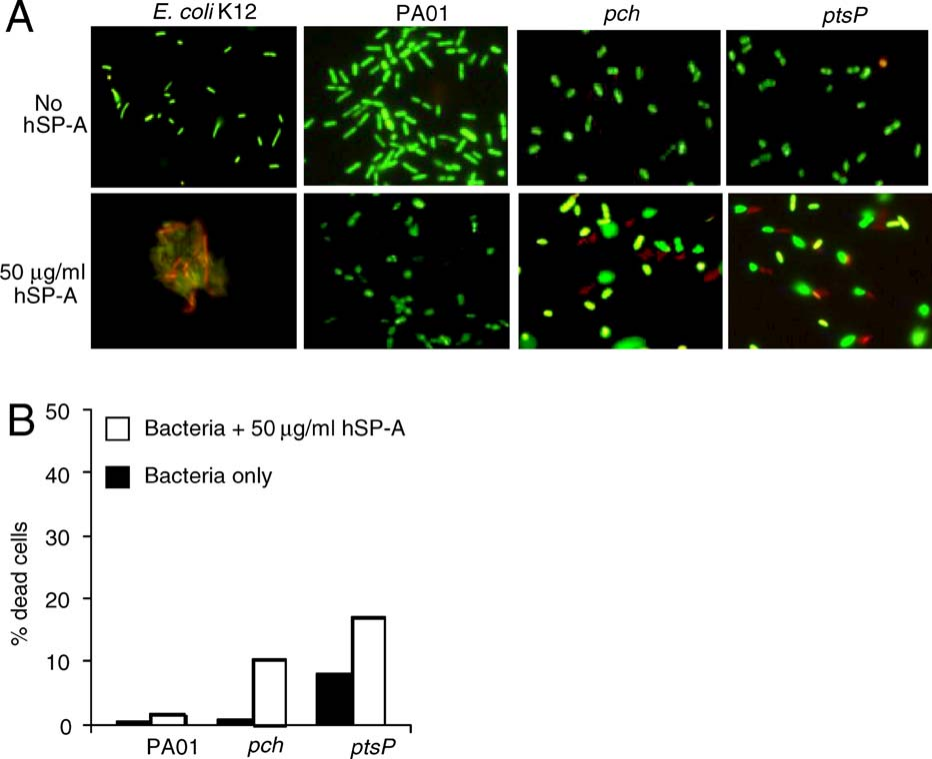
SP-A-Mediated Membrane Permeabilization Directly Kills the *pch* and *ptsP* Mutants (A) Live/Dead staining was performed on *E. coli* K12, PA01, *pch,* and *ptsP* cells, following 1 h membrane permeabilization with 100 μg/ml SP-A. Green-stained cells are alive whereas red-stained cells are dead. (B) Enumeration of live or dead bacteria following a 1 h exposure to hSP-A. At least 600–1,000 bacterial cells were counted under a fluorescence microscope. The mean of two experiments is shown.

We next determined if SP-A mediated permeability kills STM mutants. Live/dead staining, based on exclusion of propidium iodide from the live cells, was performed on bacterial cells following membrane permeability assays. Viable cells are stained green while dead cells are stained red. As shown in Figure 6A, SP-A did not kill wild-type PA01 (green stained). In contrast, mixtures of green- and red-stained cells of *pch* and *ptsP* were visible following treatment with SP-A. Approximately 11% and 9 % of *pch* and *ptsP* cells, respectively, were killed (Figure 6B) within 60 min, in comparison to about 40% of *E. coli* K12 (unpublished data). In addition, in contrast to the robust SP-A-induced aggregation of *E. coli* K12 (Figure 6A), SP-A did not aggregate *P. aeruginosa* PA01, *pch,* or *ptsP.* These results suggest that the loss of intact LPS (i.e. rough LPS), or the inability to synthesize salicylate and phosphoenolpyruvate-protein-phosphotransferase, renders the cells vulnerable to membrane permeabilization and killing by SP-A. Furthermore, bacterial aggregation does not play a role in preferential clearance of the *pch* and *ptsP* mutants.

### Complementation of the *pch* and *ptsP* Mutants Restores Their Resistance to SP-A-Mediated Membrane Permeabilization

**Figure 7:**
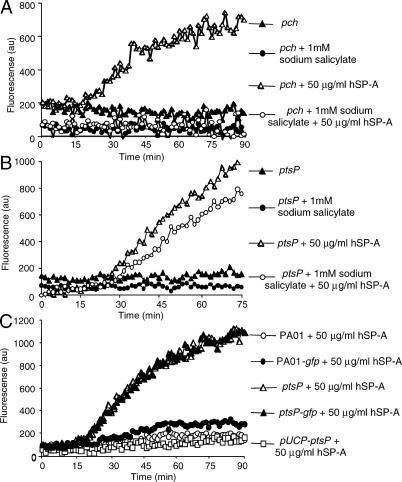
Complementation Studies of SP-A-Sensitive Mutants *pch and ptsP* (A) Culturing *pch* bacteria in LB supplemented with sodium salicylate restored its resistance to SP-A mediated membrane permeabilization. (B) In control experiments, we demonstrated that the enzymatic activity of bacterial phosphatases in *ptsP* bacterial cells was not inhibited by 1 mM sodium salicylate. (C) Provision of the wild-type *ptsP* gene on the pUCP19 *in trans (pUCP-ptsP)* restores the ability of STM mutant *ptsP* to resist SP-A mediated membrane permeabilization. In contrast, *ptsP* mutant expressing GFP gene on the pUCP19 *(pstP-gfp)* is permeabilized to similar levels as *ptsP*.

The susceptibility of STM mutants to SP-A mediated membrane permeabilization can be exploited for complementation analyses. As the *pch* mutant is disrupted in a gene required for salicylate biosynthesis, we performed complementation analysis by growing the *pch* mutant bacteria in the presence of 1 mM sodium salicylate. As shown in Figure 7A, the *pch* mutant bacteria grown in LB were susceptible to SP-A-mediated membrane permeabilization. In contrast, the *pch* mutant bacteria grown in LB supplemented with 1 mM sodium salicylate were fully resistant to membrane permeabilization by SP-A. We further examined whether the increased susceptibility to SP-A-mediated membrane permeability was due to the inability *pch* bacteria to synthesize the siderophore pyochelin. The *pch* bacteria cultured in pyochelin-supplemented LB or iron-deficient minimal medium were still membrane permeabilized by SP-A (unpublished data). Collectively, these results suggest that the increased susceptibility to SP-A-mediated membrane permeability in the *pch* bacteria is due to their inability to synthesize salicylate, or both salicylate and pyochelin. However, we cannot completely rule out the possibility that pyochelin may be involved in resistance to SP-A-mediated membrane permeability until we have exhausted all the growth conditions and optimal pyochelin concentrations for the assays.

To rule out the possibility that 1 mM sodium salicylate may inactivate the enzymatic activity of the periplasmic phosphatases that cleaves the impermeant substrate, ELF97, in our permeability assay*,* we also tested *ptsP* mutant bacteria cultured in the presence or absence of 1 mM sodium salicylate. The *ptsP* mutant bacteria were permeabilized to the same extent, regardless of whether they were grown in the presence or absence of 1 mM sodium salicylate (Figure 7B), suggesting that salicylate did not inactivate phosphatase. These results suggest that the loss of *pch* gene function results in susceptibility of *P. aeruginosa* to clearance by SP-A by rendering the organism susceptible to membrane permeabilization.

We next tested if providing the wild-type *ptsP* gene *in trans* to the *ptsP* STM mutant bacteria could restore the resistance to SP-A-mediated membrane permeabilization. As shown in Figure 7C, the resulting strain, *pUCP-ptsP* was as resistant to SP-A-mediated membrane permeabilization as the wild-type PA01. In contrast, providing an irrelevant *gfp* gene in the *ptsP* bacteria *(ptsP-gfp)* did not restore the resistance of *ptsP* STM mutant to SP-A, nor did it alter the resistance or susceptibility of the parental wild-type PA01 (PA01-*gfp*) to permeabilization by SP-A (Figure 7C). These results suggest that *ptsP* gene function protects *P. aeruginosa* from membrane permeabilization by SP-A.

### LPS Profiles of STM Mutants

Previous studies in *E. coli* [6] and *Bordetella pertussis* [19] have demonstrated that the structures of LPS on the surface of bacterial cells determine resistance to SP-A-mediated aggregation and membrane permeabilization. *P. aeruginosa* LPS mutants are also sensitive to SP-A-mediated membrane permeability, but are not aggregated by the collectin (Figure 5A; unpublished data). To examine if the *pch* and *ptsp* mutants have LPS defects, we compared the LPS profiles after separation by SDS-PAGE and silver staining. The LPS from wild-type PA01 and the *pch* mutant were indistinguishable (unpublished data). However, the two major LPS core bands of the *ptsP* mutant showed a reversal in relative abundance compared with the PA01 (unpublished data). Thus, there may be changes in LPS, at least in terms of the proportion of two different cores made. More detailed molecular analyses will be required to determine if LPS modification occurs within *pch* and *ptsP* mutants.

## Discussion

The genome-wide mutagenesis scheme called STM was originally developed to identify novel vaccine and antibiotic targets [9]. STM relies on the ability of the pathogen in question to replicate in vivo as a mixed population, and allows the identification of the virulence genes whose mutant phenotypes cannot be trans-complemented by other virulent strains present in the same inoculum. Hundreds of virulence factors and putative drug targets have been identified by STM in at least 20 different bacterial and fungal pathogens [21]. In all of these screens, a pathogen of interest is mutated by transposon or suicide vector-based insertional strategies. The vectors are each individually tagged with a DNA oligonucleotide to allow mutant identification after selection within the host. Attenuated insertion mutants that are killed by host defense mechanisms such as exposure to SP-A, will fail to amplify from lung homogenates harvested 16 h after intratracheal inoculation (Figure 2A). Comparative STM screening using wild-type and genetically altered mice, as presented herein, represents a novel application of the STM technique. This approach allows the identification of bacterial determinants targeted by specific components of host defense, rather than the entire immune system as a whole. Thus, in contrast to conventional STM screens in wild-type mice, which yield an attenuated mutant recovery rate of 2%–10% [21], our comparative STM screen to identify factors that protect bacteria against a specific host innate immunity protein, SP-A, had a yield of only 0.12%. As STM screens, by definition, only recover mutations in non-essential genes, another reason for detecting few mutants in this screen is that these genes involved in resistance to SP-A may be essential.

In this study, we have employed STM technology to identify bacterial factors that are required for *P. aeruginosa* strain PA01 to confer resistance to SP-A. Both mutants were susceptible to SP-A-mediated membrane permeabilization, confirming that disruption of membrane integrity is one of the mechanisms by which SP-A mediates bacterial clearance. The degree of bacterial membrane permeabilization of the STM mutants by SP-A was comparable to that of *E. coli* K12, which has rough LPS [6]. Based on the results of in vitro assays, macrophages and neutrophils do not seem to play important roles in preferential clearance of the *pch* and *ptsP* mutants. However, we cannot exclude the possibility that phagocytosis contributes to the differential clearance of these two mutants in vivo.

Thus far, we have not recovered STM mutants that are susceptible to SP-A-mediated opsonization, or mutants that are disrupted in the genes required for LPS biosynthesis, which play critical roles in resistance to SP-A-mediated membrane permeability. The lack of recovery of opsonization-sensitive and LPS mutants are not particularly surprising, considering that we screened only approximately 30% of the 5,570 mutants required for 1 × genome coverage (http://www.pseudomonas.com). Additional screenings are being carried out to catalog other *P. aeruginosa* factors conferring resistance to SP-A.

The mechanisms by which mutations in *pch* and *ptsP* genes result in susceptibility to SP-A-mediated clearance are briefly discussed below. The *pchDCBA* gene encodes enzymes involved in the synthesis of iron-chelating siderophores salicylate and pyochelin [12–14]. In another closely related pathogen, *Burkholderia cepacia*, salicylate is a major siderophore [22]. Salicylate has also been reported to chelate magnesium (Mg^2+^) and calcium [23]. Thus, it is possible that *P. aeruginosa* requires salicylate and its downstream product pyochelin to acquire cations. Divalent cations, especially Mg^2+^, are required to structurally stabilize LPS on the outer membrane. The recently resolved structure of SP-A protein indicates that there are two metal binding domains within SP-A, which are most likely occupied by calcium [24]. Thus, SP-A may sequester cations from alveolar lining fluid and result in a growth disadvantage for the salicylate-deficient *pch* mutant. Furthermore, inability to acquire cations may potentially alter the expression, stability, and modification of various transmembrane proteins and glycolipids by enzymes that require cations as cofactors. Thus, we speculate that the *pch* mutation may have contributed to its increased sensitivity to SP-A-mediated membrane permeabilization through direct or indirect effects on LPS integrity and membrane stability.

The *ptsP* gene encodes phosphoenolpyruvate-protein phosphotransferase EI^Ntr^ [15], a transcriptional regulator of RpoN-dependent operons [25]. RpoN is known to regulate the expression of variety of virulence factors in pathogenic bacteria, including the upregulation of the *rfaH* gene, which is required for increased production of the O-antigen of LPS in *Salmonella enterica serovar Typhi* during the stationary phase of growth [25]. The additional O-antigen production during the stationary phase of bacterial growth may be necessary for resistance to SP-A-mediated membrane permeabilization. *PtsP* also coordinates carbon and nitrogen metabolism and is required for virulence in *P. aeruginosa,* by an unknown mechanism [26]. Mutational inactivation of the *Azotobacter vinelandii ptsP* ortholog affects lipidic poly-hydroxybutyrate accumulation within its cytoplasm as a carbon source for long-term starvation survival [27]. Interestingly, poly-hydroxybutyrate is found in the plasma membranes of *E. coli* complexed to calcium polyphosphate, and forms divalent cation-selective channels [28]. Thus, increased sensitivity to SP-A-mediated membrane permeabilization in *ptsP* mutants may be caused by its reduced ability to transport divalent cations necessary to maintain LPS and outer membrane integrity.

Our results suggest that the killing of *P. aeruginosa* PA01 and STM mutants is more robust in vivo than in vitro. One potential explanation for this discrepancy is that our in vitro membrane permeability conditions do not accurately model the environment in the lung. In addition, it is possible that multiple factors contribute to the killing of *P. aeruginosa* including SP-A, SP-D, lactoferrin, β-defensins, and other antimicrobial peptides and proteins [29,30]. We are currently examining whether these peptides act synergistically or cooperatively with SP-A in clearance of *P. aeruginosa*.

Decreased SP-A levels have been found in several respiratory diseases including bacterial pneumonia, adult respiratory distress syndrome [31,32], and cystic fibrosis [33–35]. There is some in vitro evidence to suggest that decreased SP-A and SP-D levels in some of these disease states result from degradation by neutrophil proteases of the host [36,37], or by proteases of bacterial origin [38]. These proteases include bacterial elastases, and neutrophil-derived cathepsin G, elastase, and proteinase-3. The reduced levels of SP-A and SP-D in these lung diseases may contribute to increased susceptibility to infections by variety of microbial pathogens. Degradation of SP-A by *P. aeruginosa* proteases may also explain our failure to develop informative in vitro STM screens (unpublished data).

Antibiotic-resistant *P. aeruginosa* is an emerging clinical problem that can lead to denial for lung transplantation and death [39]. Thus, there is an added urgency to explore the use of novel, non-antibiotic-based, anti-*Pseudomonal* peptides to combat life-threatening infections with this organism. The discovery of factors governing resistance or susceptibility to SP-A may ultimately have therapeutic value.

## Materials and Methods

### Bacterial strains, media, and growth conditions.

Shaking cultures of *E. coli* DH5-α [40], SM17λpir [41], and *P. aeruginosa* strain PA01 [42] were grown in LB broth at 37 °C. When needed, LB was supplemented with 1.5% of bacto agar. Antibiotic selections were performed for *E. coli* or *P. aeruginosa* using the following concentrations: carbenicillin (50 or 300 μg/ml), kanamycin (50 or 250 μg/ml), and tetracycline (5 or 15–30 μg/ml).

### Animal husbandry.

The C3H/HeN SP-A^−/−^ mice were developed as previously described [6,7]. C3H/HeN control (SP-A^+/+)^ mice were purchased from Charles River Laboratory (Boston, Massachusetts, United States). All infections were performed intranasally with age-matched, 6-wk-old mice. Animals were housed in positively ventilated microisolator cages with automatic recirculating water, located in a room with laminar, high-efficiency, particle accumulation-filtered air. The animals received autoclaved food, water, and bedding. Mice used in experimental procedures were handled in accordance with protocols approved by the Institutional Animal Care and Use Committee at University of Cincinnati College of Medicine.

### Comparative screening of the *P. aeruginosa* STM library in SP-A^+/+^ and SP-A^−/−^ mice.

The *P. aeruginosa* PA01 STM library and screening methods were previously described [11]. An adapted acute mouse lung pneumonia model of infection was employed, where adult mice rather than the infant mice were used [16]. In earlier experiments, we detected no significant difference in SP-A expression by ELISA assays at 8, 16, and 24 h in SP-A^+/+^ mouse lungs infected with bacteria (1 × 10^7^ cells) from a representative pool of STM library (unpublished data). We chose the 16 h time point to screen the STM library because it gave us the most reproducible PCR amplification of DNA tags from mutants, with ≥ 10,000 colony forming units (CFU) still recoverable from the SP-A^+/+^ mice (unpublished data). In contrast, prolonged infection (≥ 36 h) with 1 × 10^7^ bacteria from STM pool frequently killed SP-A^−/−^ mice (unpublished data). Briefly, SP-A^+/+^ and SP-A^−/−^ mice [6,7] were anaesthetized using isofluorane, and were intranasally inoculated with 50 μl (1 × 10^7^ cells) of each individual STM pool. After 16 h, lungs were removed from sacrificed mice, and homogenized tissues were plated on LB agar plates. At least 10,000 bacterial colonies recovered after in vivo selection were used for multiplex PCR as described previously [11]. Twenty sets of 72 mutants (total = 1,440) were pooled and screened. An additional 240 mutants previously shown to be attenuated in a chronic model of rat lung infection [11] were also screened. The mutants that were differentially cleared by SP-A during the in vivo passaging were subsequently retested by single PCR in bacterial cells recovered from three separate mice.

### In vivo competitive and single infection assays.

For competition assays, mouse lungs were inoculated intranasally with bacterial cells (1 × 10^7^ in 50 μl) composed of a 1:1 ratio of wild-type PA01 and its isogenic STM mutants. Infected lungs were recovered 16 h after infection for bacterial load determination. The competitive index (CI) is defined as the output ratio of mutant to wild-type bacteria divided by the input ratio of mutant to wild-type bacteria [16,17]. Thus, if a mutant strain is less competitive than the parental strain from which it was derived, a CI value of < 1 will be achieved, indicating that the mutant is attenuated. Single organism inoculations with individual bacterial strains were performed by the intranasal route in SP-A^+/+^ and SP-A^−/−^ mice (group of six). Attenuation was defined as the log_10_ difference in CFU between wild-type and mutant bacteria recovered from lung tissue 16 h after inoculation.

### Mutant cloning and bioinformatics analysis.

Cloning of putative STM mutants were performed as previously described [11]. The DNA sequences were analyzed with MacVector or DNA Star software. Sequence data was compared with the database available for *P. aeruginosa* at http://www.pseudomonas.com.

### Non-polar deletion and complementation of the *ptsP* mutant.

Non-polar, in-frame deletion of *ptsP* was generated by PCR: 0.802 and 1.155-kb 5′ and 3′ segments were amplified from target PA01 genomic DNA, and each amplicon, which included an engineered restriction site, was ligated into pEX18Ap [43] to produce replacement plasmids. Inframe deletion mutants were generated in PA01 via homologous recombination by sucrose resistance selection, and confirmed by hybridization and PCR analysis (unpublished data). To complement the *ptsP* mutation, a 4.5-kb DNA fragment containing both the promoter and the *ygdP-ptsP-PA0338* operon (Figure 2C) was PCR-amplified from the wild-type parental strain PA01 using the Expand Long Template PCR System (Roche Diagnostic, Basel, Switzerland), and cloned into *E. coli-P. aeruginosa* shuttle plasmid pUCP19 [44] to obtain pUCP19*-ptsP*. The cloned fragment was sequenced to rule out any mutation that was introduced during the amplification. The pUCP19*-ptsP* was introduced into *P. aeruginosa* STM mutant strain *ptsP* by heat shock. Carbenicillin-resistant transformants were selected, and verified by Southern hybridization and PCR methods (unpublished data). Complementation was assessed by resistance to SP-A-mediated membrane permeabilization.

### Complementation of the *pch* STM mutant.

Complementation of the *pch* mutant was performed by culturing the bacteria into stationary phase in LB supplemented with 1 mM sodium salicylate.

### Pyochelin extraction and detection.

Pyochelin production in *P. aeruginosa* was analyzed using the method previously described [45]. M63 minimal medium (5 ml) was inoculated with fresh colonies of individual *P. aeruginosa* strains and incubated at 37 °C for 24 h with shaking. A fresh 30 ml of M63 supplemented with 0.5% w/v casamino acids was then inoculated with 0.3 ml of starter culture, and incubated for 36 h at 37 °C with shaking. The culture was then centrifuged at 13,000 rpm for 10 min in 1.5 ml aliquots in a microcentrifuge. The supernatants were removed into a 50 ml polypropylene screw-capped centrifuge tube, pooled, adjusted to (pH 2.0) with 1M HCl, using universal indicator paper to estimate pH, and filter sterilized through a 0.22 μM membrane. Pyochelin was extracted by the addition of 0.4 volumes of ethyl acetate, followed by vigorous vortexing. The two phases were separated by centrifugation at 2,000 rpm for 5 min in a bench top centrifuge, and the upper, organic phase was removed to a separate tube. Pyochelin extract was concentrated by rotary evaporation at 40 °C in 100 ml round bottomed flasks, and the residue was dissolved in 300 μl of methanol. Pyochelin extracts (10 μl) was separated by TLC (silica gel 60, plastic backed, from Sigma, St. Louis, Missouri, United States), using acetone:methanol:0.2M acetic acid (5:2:1) as a development solvent. The chromatography tank was left to stand for several minutes with its lid on to allow the air inside to become saturated with solvent vapor. Pyochelin, which is fluorescent, naturally occurs as two stereoisomers. When visualized under a UV transilluminator, it is revealed as two green fluorescent bands. Pyochelin standard was generously provided by Dr. J. M. Meyer (Université Louis Pasteur).

### Purification of human SP-A.

Human SP-A (hSP-A) was purified from lung washings of patients with the lung disease alveolar proteinosis, as previously described [6]. SP-A preparations were re-suspended in 5 mM Tris, 150 mM NaCl. SP-A preparations were determined to contain 140–190 pg of LPS/μg of SP-A by the Limulus Amebocyte Lysate (BioWhittaker, Rockland, Maryland, United States).

### Assays of *P. aeruginosa* permeability.

The effect of the SP-A on *P. aeruginosa* cell wall integrity was assessed by determining permeability to a phosphatase activity substrate, Enzyme-Labeled Fluorescence 97 (ELF-97, Molecular Probes, Eugene, Oregon, United States) as described [7]. SP-A was incubated with 1 × 10^8^ bacterial cells/ml in 100 μl of 5 mM Tris, 150 mM NaCl for 15 min at 37 °C, and 100 μM ELF97 phosphatase substrate was added. Fluorescence was measured at excitation and emission wavelengths of 355 and 535 nm, respectively, for a period of 60–90 min.

### In vitro macrophage and neutrophil phagocytosis assays.

Freshly lavaged mouse macrophages (~ 5 × 10^5^ from 3 SPA^+/+^ mice) were cultured on chamber plastic culture slides, coated with 5% poly-D-lysine with 200 μl RPMI (Dulbecco's, containing 2.5 mg/L gentamycin and 0.1% BSA) and used immediately. Macrophages were allowed to adhere for 2 h at 37 °C in 5% CO_2_. Live GFP-expressing PA01 or isogenic STM mutants in RPMI were opsonized with or without 25 μg/ml human SP-A for 1 h at 37 °C with rotation. The medium was removed from each well and replaced with 250 μl of opsonized PA01 or STM mutants, and incubated for 1 h at 37 °C in 5% CO_2_ at a ratio of 100 bacteria to 1 macrophage. Chamber slides were washed three times with PBS containing 1 mM CaCl_2_. Extracellular bacteria were quenched with crystal violet (0.8 mg/ml). Following two additional washes, cells were fixed with 1% paraformaldehyde in PBS plus 1 mM CaCl_2_ for 10 min, and stained with Evans blue for 2 min. The percentage of macrophages with engulfed bacteria was quantified under a phase contrast fluorescence microscope. At least 200 macrophages were counted. Human neutrophils were purified and cultured as previously described [46]. Neutrophil phagocytosis was performed as described for macrophages.

### LIVE/DEAD bacterial staining.

The viability of SP-A exposed bacteria was determined by LIVE/DEAD Baclight^TM^ Bacterial Viability Kit (Molecular Probes). Briefly, stationary phase *E. coli* or *P. aeruginosa* bacteria were washed three times with 5 mM Tris, 150 mM NaCl, and adjusted to an of OD_600_ = 1.0. A 100-μl aliquot of the cells was added to culture tubes containing 100 μl of SP-A (100 μg/ml) or 100 μl of 5 mM Tris, 150 mM NaCl, as a control. The mixtures were incubated at 37 °C, 300 rpm for 1 h, and stained according to the instructions provided by the supplier. The viability of the cells was checked under a fluorescence microscope. At least 1, 000 cells were counted.

### LPS preparations.

LPS was isolated from *P. aeruginosa* by acetone/phenol extraction [47]. LPS samples were size-fractionated on 4%–12% Bis-Tris PAGE gels (NuPAGE Novex Gels; Invitrogen, San Diego, California, United States) and stained with silver.

### Statistical analysis.

Statistical analysis was performed using the Student's *t*-test and one-way analyses of variance (ANOVA). A significant difference was considered to be *p* < 0.05.

## Abbreviations

CFU: colony forming unit
CI: competitive index
GFP: green fluorescence protein
LB: Luria broth
LPS: lipopolysaccharide
RFLP: restriction fragment length polymorphism
SP-A: surfactant protein A
SP-D: surfactant protein D
STM: signature-tagged mutagenesis
TLC: thin layer chromatography
